# Synergistic Approach toward Erbium-Passivated Triple-Anion Organic-Free Perovskite Solar Cells with Excellent Performance for Agrivoltaics Application

**DOI:** 10.1021/acsami.1c23476

**Published:** 2022-01-31

**Authors:** M. Bilal Faheem, Bilawal Khan, Chao Feng, Syed Bilal Ahmed, Jiexuan Jiang, Mutee-Ur Rehman, W. S. Subhani, M. U. Farooq, Jinlan Nie, M. M. Makhlouf, Quinn Qiao

**Affiliations:** † Institute of Fundamental and Frontier Sciences, University of Electronic Science and Technology of China (UESTC), Chengdu 610054, P.R. China; ‡ Department of Materials Science and Engineering, City University of Hongkong, Hongkong SAR 999077, China; § School of Physics, University of Electronic Science and Technology of China, Chengdu 610054, China; ∥ Department of Sciences and Technology, Ranyah University College, Taif University, P.O. BOX 11099, Taif 21944, Saudi Arabia; ⊥ Energy Generation and Storage Lab, Department of Mechanical and Aerospace Engineering, Syracuse University, Syracuse, New York 13244, United States

**Keywords:** organic-free Perovskite solar cell, pre-heated solution dropping, stabilized power output, initial radiative efficiency, agrivoltaics

## Abstract

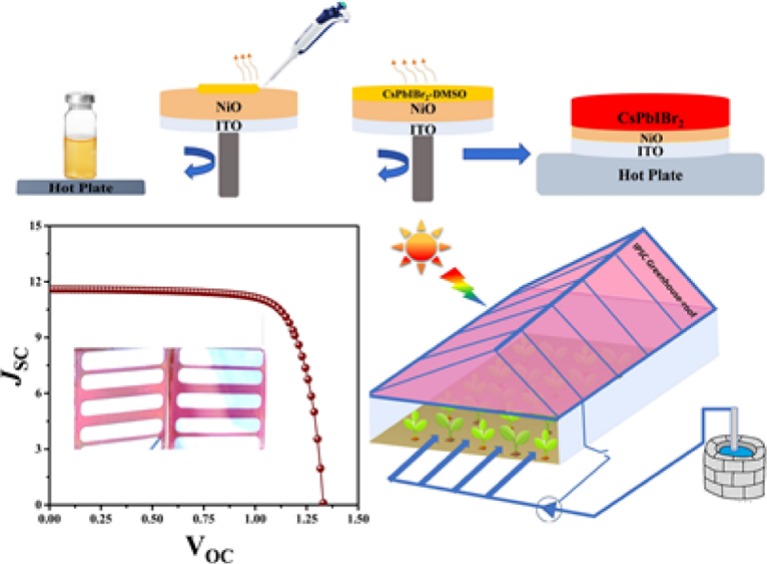

All-inorganic perovskite solar cells (IPSCs) have gained massive attention due to their less instability against common degradation factors (light, heat, and moisture) than their organic–inorganic hybrid counterparts. Inorganic perovskites bear a general formula of CsPbX_3_ (X = Cl, I, Br). The mixed halide CsPbIBr_2_ perovskite possesses an intermediate band gap of 2.03 eV with enhanced stability, which is still available for photovoltaic applications and the research focus of this work. We present a synergistic approach of pre-heated solution dropping with inorganic additive inclusion to deposit the organic-free triple anion CsPbIBr_2_ PSC. Erbium (Er)-passivated triple-anion CsI(PbBr_2_)_0.97_(ErCl_3_)_0.03_ IPSCs with inorganic carrier selective layers (CTLs), that is, organic-free, are fabricated with enhanced carrier diffusion length and crystalline grain size while lessening the grain boundaries near perovskite active layer (PAL)-bulk/carrier selective interfaces. As a result, the trap-state densities within the perovskite bulk were suppressed with stabilized CTL/PAL interfaces for smooth and enhanced carrier transportation. Therefore, for the first time, we contradict the common belief of *V*_OC_ loss due to halide segregation, as a nice *V*_OC_ of about 1.34 V is achieved for an organic-free IPSC through enriching initial radiative efficiency, even when halide segregation is present. The optimized organic-free IPSC yielded a power conversion efficiency of 11.61% and a stabilized power output of 10.72%, which provides the potential opportunity to integrate into agrivoltaics (AgV) projects.

## 1 Introduction

Organo-metal halide perovskite solar cells (PSCs) have brought about a research revolution in the field of photovoltaics (PVs), as their power conversion efficiencies (PCEs) have skyrocketed compared to conventional Si-based solar cells. PSCs have enabled a swift rise in (PCE) for lab-scale devices from 3.8^1^ to 25.6%^2^ just in 11 years of research efforts, and their commercialization is already under progress. Despite the appealing performance at the laboratory scale, the fragility of organic moiety against temperature, illumination, and moisture mishmashes resulted in meager device performance, which is the only Achilles’ heel precluding them from their potential commercialization.^3−5^ To solve instability issues, ever-growing attention has been paid toward the development of inorganic PSCs (IPSCs) (CsPbX_3_, where X = Br, I) having exceptional compositional and thermodynamic stability.^6−9^ In general, the IPSCs comprise CsPbBr_3_,^10,11^ CsPbI_2_Br,^12,13^ CsPbIBr_2_,^14,15^ and finally, CsPbI_3_.^7,16^ Among them, CsPbI_3_ and CsPbI_2_Br possess relatively narrower band gaps of 1.73 and 1.92 eV and the highest PCEs of 20.37^16^ and 17.45%,^17^ respectively, but they have poor immunity to sustain their perovskite phase against room ambience and convert it to a non-perovskite orthorhombic (non-photoactive) phase.^12,18−20^ CsPbBr_3,_ on the other hand, has very nice intrinsic (phase) and environmental stability, its large band gap (2.3 eV) results in insufficient light absorption and lower PCE.^21,22^ Besides its deployment as a perovskite active layer (PAL) into PSC, CsPbBr_3_ has proved to be beneficial as an interlayer to stabilize the ETL/PAL interface. The nanocrystal of CsPbBr_3_ was reported to modify and passivate the SnO_2_/MAPbI_3_ interface to strengthen the oriented migration of photogenerated carriers across the interface, and the optimized PSC yielded a stabilized PCE exceeding 20%.^23^ Moreover, Br-rich IPSCs can generate a high open-circuit voltage (*V*_OC_),^24,25^ which is beneficial to develop tandem PSCs and catalytic devices for water splitting.^26−28^ Wide band gap perovskites have the potential to enhance the stability of the bottom cell against ultraviolet radiation and act as a filter in tandem cell configurations.^25^ The CsPbIBr_2_ perovskite has both an intermediate band gap (2.06 eV) and excellent thermal stability to trade-off the absorption ability and intrinsic stability. Nevertheless, the PCEs of Br-rich IPSCs are far from their maximum theoretical efficiencies, which is caused by their undesirable absorption threshold and non-radiative recombination.^29,30^ The initial radiative efficiency (IRE) is lowered by phase segregation, which leads to trap states and polaron formation near the carrier selective layer (CTLs) interfaces and *E*_loss_ (*E*_loss_ = *E*_g_ – *eV*_OC_) within the perovskite bulk, limiting the overall *V*_OC_. Several studies have been reported to reduce the *E*_loss_, including interface- and compositional-engineering and organic additive inclusion, and so forth.^13,31,32^ Organic additives were reported as a benign strategy to suppress the trap states,^33^ whereas high annealing temperatures required in the crystallization process of IPSCs limit their deployment into inorganic perovskites.^34^ Therefore, the partial substitution of extrinsic heteroatoms at Cs- and Pb-sites of the perovskite structural cage is a widely adopted approach to modulating the perovskite properties. The conduction band minimum (CBM) of the perovskite is mainly originated from the p-orbital of Pb^2+^, and substitutional doping at the Pb site could tune the optoelectronic properties of the resultant perovskite. A dopant should have a similar valency (+1, +2, and +3), suitable electronegativity, and ionic radii as the threshold for respective site doping in a crystal lattice.^35,36^ In most studies regarding heteroatom doping, the Pb site has been a target for substitution to suppress mixed halide segregation and enhance carrier lifetimes. Many groups have synthesized CsPbIBr_2_ IPSCs through various strategies,^37^ as Shao et al. reported the functional doping of Cu^2+^ in CsPbIBr_2_ with appropriate content to enhance the overall device PCE by up to 10.4%. The improved performance is attributed to nice morphology and fluent carrier transportation through CTL by a fine energy-level alignment.^38^ Tang et al. synthesized the (NiCo)_1–*y*_Fe_*y*_O_*x*_ nanoparticles-decorated graphene oxide as a p-type carrier booster within CsPbIBr_2_ IPSCs, and the device presented a higher PCE of 10.95% with a *V*_OC_ of 1.29 V.^39^ Gao and Meng presented crystal interface passivation for CsPbIBr_2_ IPSC with polyethyleneimine. The optimized PSC device yielded a high PCE of 11.3%.^40^ Most recently, carbon electrode-based CsPbIBr_2_ PSC was reported with an optimized bulk heterojunction layer inserted between the PAL and counter electrode. The bulk heterojunction layer was composed of a poly (3-hexylthiophene-2,5-diyl) and [6,6]-phenyl methyl C61 butyric acid methyl ester (P3HT/PCBM) that enhanced the light absorption capability of PAL, optimized the carrier transport dynamics, and inhibited dark recombination while yielding an overall PCE of 11.54%.^41^ In most reports regarding IPSCs, organic CTLs are still included, which is a bigger reason to worry about the narrative of improved overall device performance.

We report the fabrication of the organic-free triple-anion CsPbIBr_2_ IPSC with an organic-free device architecture (ITO/NiO/CsPbIBr_2_/Nb_2_O_5_/Ag) through the synergistic approach of pre-heated solution dropping together with inorganic additive (ErCl_3_) inclusion. The PALs of CsPbIBr_2_ and CsI(PbBr_2_)_0.97_ (ErCl_3_)_0.03_ are deposited through pristine and optimized pre-heating temperatures with ErCl_3_ inclusion to harness improved crystallinity and morphology. Remarkably, PALs derived from ErCl_3_ doping present micrometer-sized crystalline grains and enhanced carrier lifetimes with reduced trap-state density. We surmised that the heteroatom doping of the Er salt not only enhances the bulk properties but also improves carrier transportation across the CTL/PAL interface. Moreover, the optimized organic-free PIN (ITO/NiO/CsI(PbBr_2_)_0.97_ (ErCl_3_)_0.03_/Nb_2_O_5_/Ag) device yielded a nice PCE of 11.61% (which is 60% higher compared to its pristine counterpart’s PCE of 7.28%) with a *V*_OC_ of 1.34 V, a fill factor (FF) of 70.5%, and a stabilized power output (SPO) of about 10.72%. The overall organic-free PSC retained 86% of its initial efficiency after aging for 688 h under continuous 1 sun illumination. To the best of our knowledge, this is the first-ever report for the organic-free CsPbIBr_2_ IPSC.

Additionally, we fabricated the semi-transparent IPSC based on the same synergy with a slightly decreased photocurrent, and the device achieved a PCE of 10.71%. Moreover, we present a devised AgV design based on the absorption region of wide-band gap CsPbIBr_2_. The optimized semi-transparent device utilizes merely <20% photons in the solar spectrum, resulting in an exceeding PCE, whereas the remaining photons are adequate to bring about photosynthesis and greenhouse heating.

## 2 Results and Discussion

**Figure 1:**
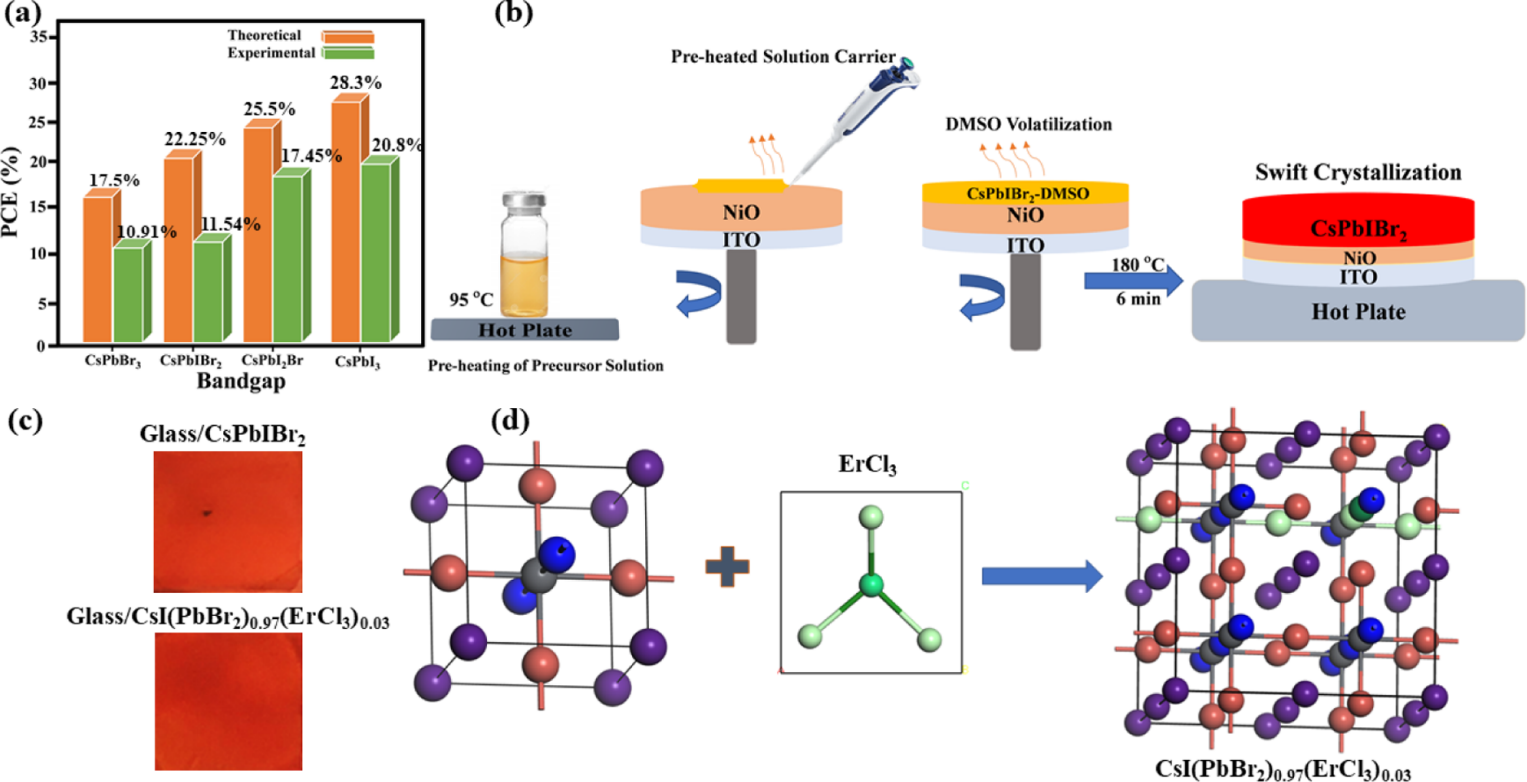
(a) Statistics of CsPbX_3_ PSCs PCE. (b) Synthesis schematics for the synergistic approach of pre-heating and additive inclusion. (c) Original photographs taken for pristine and synergistically derived PALs. (d) Schematic illustration of substitutional occupation of Er and Cl into a perovskite structural cage.

Previous reports have described that Br-rich IPSCs are still far from their theoretical PV parameters, such as device PCE. Figure 1a shows the statistical view comparing theoretical and practical PCEs for CsPbX_3_-based PSCs attained to date.^42^ Hot solution dropping as an optimized protocol can thermodynamically sustain the supersaturation of the precursor solution, which further helps improve the precursor solution’s entropy with control over nucleation and crystallization of PAL. Figure 1b illustrates the schematics for pre-heated solution dropping onto an ITO/NiO substrate. The pre-heating of precursor solution in the vial was carried out while putting it on the hot plate at 95 °C for 3–4 min before the spin coating process; the reserved solution heat accelerated the effective dimethyl sulfoxide (DMSO) volatilization to derive controlled nucleation and improved PAL morphology. Moreover, the resulting PALs comprised highly crystalline micrometer (μm) grain sizes and lessened grain boundaries. We also deposited CsPbIBr_2_ PAL with a conventional spin-coating process without pre-heating to set as the pristine or control sample, and optical images in Figure 1c present that there is no noticeable change in color and transparency for synergy and pristine PALs. The crystal lattice after substitutional occupancy of Er at the Pb site and Cl at the X site is systematically illustrated in Figure 1d, as the inclusion of ErCl_3_ into the CsPbIBr_2_ structural cage does not distort the perovskite lattice and the ionic radii of additive constituents, that is, Er and Cl, are overall within the Goldschmidt tolerance factor range ***t*** 0.8 < *t* < 1.0.^35^

We characterized X-ray photoelectron spectroscopy (XPS) for pristine and synergy samples to test the microscopic interplay between perovskite’s constituent elements and to verify the effect of ErCl_3_ inclusion on CsPbIBr_2_. Figure S1a presents the schematics of XPS and the energy aspects of the emitted photoelectron through various energy levels in the perovskite bulk. For pristine PAL, the signature peaks of Cs–3d, I–3d, Pb–4f, and Br–3d could be detected obviously with no other observed peaks, as shown in Figure S1b–e (Supporting Information), indicating the pure CsPbIBr_2_ composition. Upon ErCl_3_ inclusion into CsPbIBr_2_, new peaks arose at binding energies of 168.5 and 198 eV (Figure S1f–g), which are assigned to Er–4d and Cl–2p, respectively. Er influenced the CsPbIBr_2_ lattice by substituting the Pb atom while increasing its binding energy, but there are no peaks detected solely for Pb and Er–I, which is indicative of suppressed ion migration and lessened trap-state density. Er from the ErCl_3_ additive bonded effectively with Pb, leaving no chances behind for the minority phase to rise, and so we termed it as Er passivation against Pb- or X-enhanced phase separation. The high-resolution XPS survey spectra for binding energies of different constituent elements are presented in Figure S1i, which is consistent with the previously reported studies of CsPbIBr_2_.^6,39,40^

**Figure 2:**
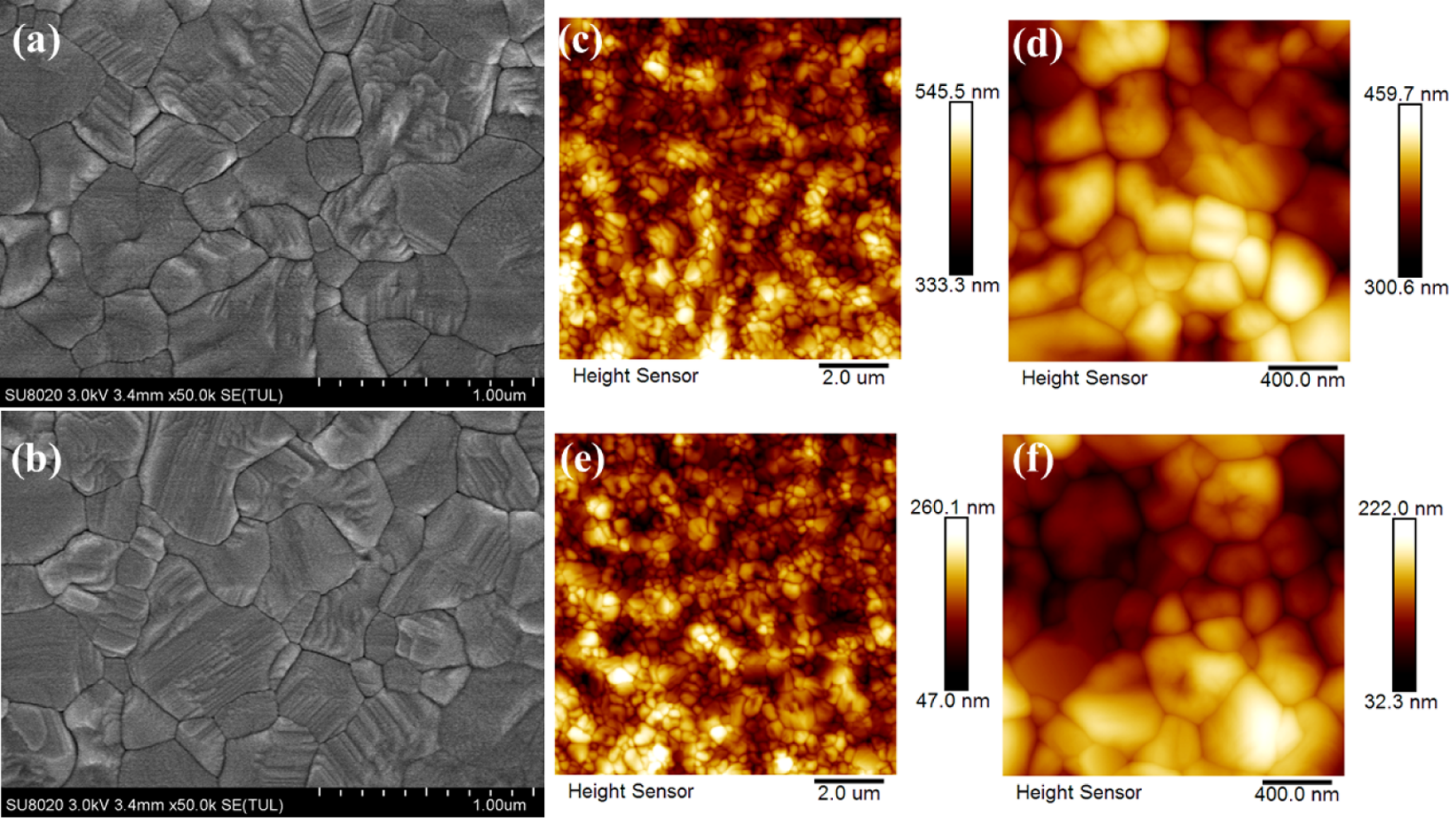
Top-view SEM image of (a) pristine and (b) optimized PALs. AFM height images with the RMS roughness of (c,e) pristine and (d,f) synergy PALs.

Surface properties like morphology and crystallinity were characterized through scanning electron microscopy (SEM) and atomic force microscopy (AFM) images together with X-ray diffraction (XRD) spectra. In Figure 2a, the top-view SEM image (PAL deposited over ITO-coated glass) for the pristine sample presents a uniform morphology with smaller crystalline grains and more grain boundaries due to heterogeneous nucleation and poor crystallization. On the other hand, SEM for the synergistic sample (Figure 2b) presents large-sized textured grains attributed to the pre-heated and additive-included solution, reducing grain boundaries and trap states within the PAL bulk. The reserved heat increased the solution entropy, thereby swiftly removing DMSO from precursor solution during spinning to yield uniform and compact films. The statistical view of the grain-size distribution for pristine and synergistic PALs is summarized in Figure S2a,b. Moreover, an improved carrier diffusion length with suppressed halide segregation is obtained through our synergistic approach. AFM images are taken to investigate the root-mean square (RMS) roughness and to verify grain boundaries’ orientation in pristine and synergy samples. Figures 2c–f present the AFM images calibrated to the corresponding height and RMS profiles at different resolutions. The pristine PAL is tested to have an RMS roughness of 19.8 nm, and it is increased for synergy PALs to 27.4 nm. A slightly higher RMS roughness is beneficial for carrier transportation across the PAL/CTL interface.^43^ It is evident that our synergistic approach influenced the growth kinetics of PAL, and the crystal structure of pristine and synergy PALs was examined through XRD, as shown in Figure S3a (Supporting Information). The prominent peaks at 15.1, 21.3, and 30.2 are assigned to (100), (110), and (200) planes of the α-CsPbIBr_2_ perovskite, respectively. The intensity of peaks for (100) and (200) planes is indicative of the perpendicular crystal growth to the substrate and is advantageous for out-of-plane carrier transport.^35,38,39^ For synergy samples, the peak intensity was boosted for (100> and (200> planes, while that for others decreased, suggesting higher crystallinity and enhanced preferential orientation, which is again attributed to lower grain-boundary scattering and intragranular defects.

Density functional theory (DFT)-based calculations are employed to investigate the conduction (CB) and valence band (VB) occupied by different constituent elements and to reveal the influence of the Er concentration on the band gap of CsPbIBr_2_. We employ the CsPbIBr_2_ perovskite with percentage doping of ErCl_3,_ that is, 0, 10, 20, and 30%. Figure S4a presents the total density of states (DOSs) for 0% doping, that is, a pure perovskite, which manifests that VB of the initial perovskite is composed of Br 4p states while CB is comprised of Pb 6p states. The VB of ErCl_3_–CsPbIBr_2_ is comprised of Br 4p and Er 4f states, but CB is still composed of Pb 6p states, as shown in Figure S4b. The halide role within the perovskite is well understood, so a minor band gap increment for Cl addition to the X site and the optimized perovskite lattice presents a band gap at 2.03 eV. The zero-state region within the elemental partial density of states (PDOSs) witnessed no peaks for any perovskite constituent elements, so the straight line separating the left (CBM) and right (VBM) regions explains the band gap of the perovskite. The calculated band gap values are less than those extracted from absorption spectra due to the quantum confinement effect and generalized gradient approximation (GGA). For doping percentages of 20 and 30%, the band gap values increased drastically as the perovskite lattice distorted beyond its tolerance factor range^35^ and showed an overall non-semiconducting behavior. In both cases of pure and doped CsPbIBr_2_, the minimal energy state in CB and the maximal energy state in VB have the same *k*-vector within the Brillouin Zone (BZ), which means that these are direct band gap semiconducting materials.

**Figure 3:**
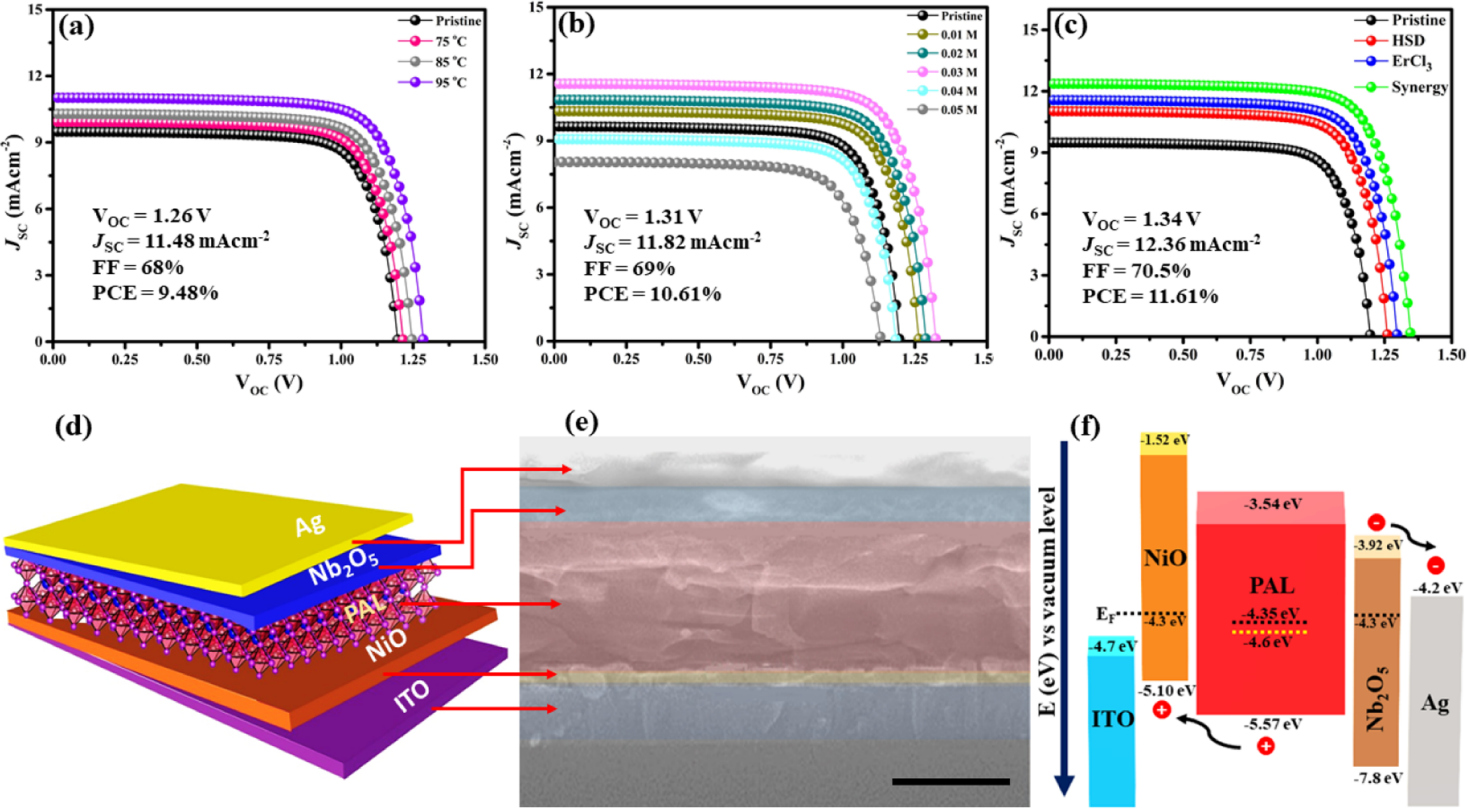
Optimized *JV* curves for (a) temperature, (b) additive inclusion, (c) synergy-IPSCs, and (d) IPSC device-schematics with different CTLs and PAL. (e) Corresponding cross-sectional SEM image (at a resolution of 300 nm) with a fake color filter verifying different CTLs and their thickness (f) energy-level diagram of the corresponding IPSC device with PAL’s Fermi level calculated through DFT results.

To explain the PV performance of CsPbIBr_2_ PSCs, we tested our IPSC devices under AM 1.5 illumination at 100 W cm^–2^. We have done a series of optimization experiments for temperature and additive concentrations, and detailed PV parameters are given in Table S1 (Supporting Information), also shown in Figure 3a–c. Noticeable improvement in *V*_OC_ is observed until the temperature or doping concentration have successfully optimized. The PAL for pre-heated solution temperature of 95 °C and 0.03 M ErCl_3_ exhibits the best PV parameters as *V*_OC_ is boosted to 1.34 V with *J*_SC_ = 12.36 mA cm^–2^ and an excellent FF of 70.5%. Figure S5 (Supporting Information) describes the statistics of PV parameters and PCE for 30 devices, which explains the reproducibility of our results. A small increment in *J*_SC_ is attributed to delocalized hole extraction from NiO–HTL and better energy-level matching between CsPbIBr_2_ and Νb_2_Ο_5_ (ETL), indicating less carrier recombination and traps beside CTL/PAL interfaces. The Cl inclusion in part at the X-site limited the *J*_SC_ enhancement due to possible band gap widening, but the CBM or VBM expansion away from the Fermi level cannot cause a prominent change in the band gap, as explained by DFT results. Figure 3d,e shows IPSC device schematics with the corresponding cross-sectional SEM image of our IPSC (indium–SnO_2_/NiO/CsI(PbBr_2_)_0.097_(ErCl_3_)_0.03_/Nb_2_O_5_/Ag), with fade color filters to better visualize the differentiation between different layers. The weak built-in potential of the NiO–HTL-based PSC device was reported as a cause of the Fermi level (*E*_F_ = −4.3 eV) shift near VBM in NiO.^44,45^ Ultra-violet photoelectron spectroscopy (UPS) is used to investigate the electronic structure of pristine and synergy PALs, as shown in Figure S6a,b (Supporting Information), which describes the UPS spectra with the secondary electron cut-off region and linear regression of the valence band maximum (VBM) onset, respectively. The calculated VBM energies are −5.57 and 5.78 eV for synergy and pristine PALs, respectively, with a similar *E*_g_ of 2.06 eV. Similarly, the work function values are confirmed to be 3.12 and 3.37 eV, respectively, for synergy and pristine samples as the calculation formulae (S1 and S2) are settled in the Supporting Information. Moreover, the Fermi level is increased by 0.25 eV for synergy PAL, which is beneficial for effective hole extraction through the PAL–HTL interface. In our work, the Fermi level [*E*_F_ = −4.35 eV (DFT) and −4.6 eV (UPS tested)] of synergy-PAL became well-tuned due to substitutional Er doping. Therefore, the overall energy-band alignment of the engineered IPSC provides fluent carrier transportation between CTLs and PAL, as shown in Figure 3f.

**Figure 4:**
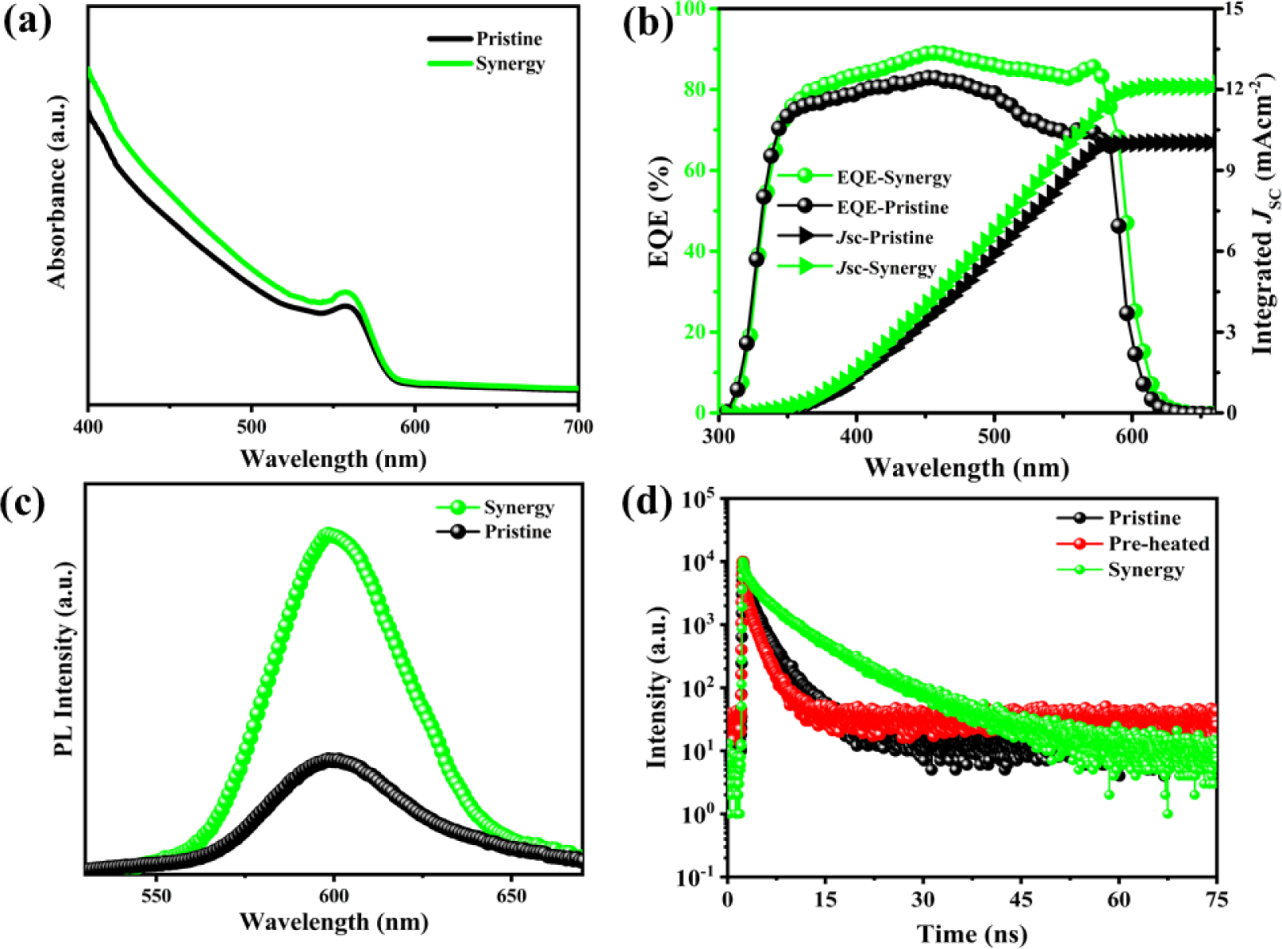
(a) UV–absorption spectra for pristine and optimized PALs. (b) IPCE spectra with EQE and the corresponding *J*_SC_ (c) PL spectra against the source-illumination intensity for pristine and optimized PALs (d) TRPL decay profiles for pristine, hot-solution dropping, and synergistic PALs.

The UV-absorption analyses were carried out for both samples: the absorption edge lay at 601 nm, which is in excellent agreement with a band gap of 2.06 eV as shown in Figure 4a, and is consistent with the reported values of CsPbIBr_2_ films.^38,39,42^ The absorption offset was slightly enhanced for optimized samples, which is attributed to chloride inclusion and effective Er passivation. The band gap has increased from 2.06 to 2.08 eV for pristine and synergy samples as calculated through UV analyses. The PV external quantum efficiency (EQE_PV_) at a specific wavelength is defined as the fraction of photons that contribute to the radiative photocurrent (*J*_SC_) of a solar cell held in a short circuit. *J*_SC_ can also be theoretically established through the overlap integral between EQE_PV_ and the solar photon flux (ϕ_AM1.5_), which is given by the following relation (1)
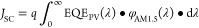
1where “*q*” is the electronic charge, “ϕ_AM1.5_” is the standard photon flux density, and “λ” is the wavelength of incident light. EQE_PV_ is tested through incident photon to current conversion (IPCE) efficiency, which provides another quantitative measurement for short-circuit photocurrent density (*J*_SC_), as shown in Figure 4b. An impressive EQE exceeding 85% in the wavelength region of 300–610 nm is obtained. There is a minute difference between *J*_SC_ values obtained through conventional *J*V measurements and IPCE, ascribed to spectral mishmashes between an IPCE source and a solar simulator. Therefore, for the pristine device, a significant decrease can be found in EQE and *J*_SC_ values. Our synergistic approach is suitable to lower the *V*_OC_ losses and to lower the dark recombination current *J*_0_; the latter investigation is not usually discussed despite being theoretically established. Experimentally, in a real-time PV device, *J*_0_ is the current that arises from thermally excited carriers, that is, the charges excited by ambient blackbody radiation (*J*_0_) are proportional to the junction-temperature id. est., ) and can be extracted from a solar cell in the dark by applying a significant reverse bias. Therefore, the radiative recombination current *J*_0,rad_ can be calculated through the following expression (2)
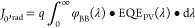
2where ϕ_BB_ is the flux of black-body radiation as a function of wavelength “λ”. *J*_0,rad_ is the sum of radiative and non-radiative recombination currents and is described through the principle of detailed balance, that is, the absorbed photon-current must equal the emitted photon-current. The *V*_OC_ yield is strongly dependent upon *J*_SC_ and *J*_0_, as explained by eq 3
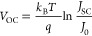
3

For an ideal solar cell, we derive the theoretics of radiative and non-radiative recombination entities as a small fraction of recombination is radiative. The non-radiative recombination current (*J*_0,non-rad_) can be calculated through the electroluminescence quantum efficiency (EQE_EL_), which always has a positive value less than unity and for which the detailed derivation can be found through equations S3 and S4 in the Supporting Information. For practical solar cells, *V*_OC_ can be formulated as eq 4
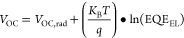
4

In the above expression, *V*_OC,rad_ is the *V*_OC_ calculated within the radiative limit of synergistically derived PAL. The sub-band gap or segregated phase-enhanced tail state absorption is virtually invisible and cannot be detected by IPCE spectra for EQE extents. Still, the occurrence of *J*_0_ is an indication that tail states are present. These tail states have no apparent influence on *J*_SC_; instead, a significant impact on *V*_OC_ was noted, so as to put it far away from theoretical limits. The absorption edge of pristine and synergy PALs shows a minor difference, which is the indication of Urbach energy within radiative limits, and the minute segregated phases are away from the detection of photoluminescence (PL) and UV spectra, which are of course, not the *V*_OC_ lowering channels as the severe *V*_OC_ deterioration would be expected only if Urbach energy exceeds **k**_**B**_**T**.^46^

The PL measurement is employed to study carrier transport and mechanistic recombination insights within the PAL bulk and beside CTL interfaces of the corresponding IPSCs. Figure 4c exhibits the steady-state PL profiles for deposited PALs, with emission peaks located at 601 nm and the intensity going higher for synergy samples, indicating that the latter has fewer defects, which is also in proximity with the UV absorption spectra and EQE absorption thresholds. Beal et al.^47^ reported that for inorganic CsPb(I_1–*x*_Br_*x*_)_3_ perovskites, phase segregation under continuous light illumination occurs when *x* > 0.4, but luckily, in the current work, no severe phase separation occurred even with the presence of triple-anion halide composition (*x* > 0.4). The solution to the said problem was devised through embedded endotaxial matrices,^48^ and besides, we claim that Cl inclusion to CsPbIBr_2_,^49^ that is, our synergistic approach to deposit triple-anion PAL, has the potential to suppress halide phase separation. If phase segregation happens inside PAL, the lower energy phase carries out the carrier funneling to raise reverse saturation current and limit *V*_OC_. However, apart from intuition, no quantitative analysis reported how halide segregation influences and limits *V*_OC_, as in our initial optimization experiments, the PAL showed phase segregation (Figure S6c) and the same PAL-based IPSC device presented a nice *V*_OC_ of 1.34 V. Likewise, the corresponding time-resolved PL (TRPL) is tested to count for carrier decay profile to compare pristine and synergy PALs deposited onto a glass substrate. Figure 4d presents that the synergistically approached PAL exhibits lower PL decay with longer carrier lifetimes (τ_avg_ = 21.76 ns) as compared to the pre-heated solution (τ_avg_ = 15.63 ns) based and pristine PAL (τ_avg_ = 9.46 ns), which is symbolic of the fact that smooth and highly crystalline PALs are deposited through our strategy, the formulae for TRPL measurements are explained in Supporting Information. Our synergistic approach presents suppressed ionic migration within PAL bulk and near grain boundaries, but the ion-migration within PAL bulk, up to a beneficial extent, is immune to perovskite’s property of self-healing.^50^ Carrier diffusion lengths and IRE are enhanced through suppressed Shockley–Read–Hall recombination. It was theoretically well established that strengthening IRE can improve *V*_OC_ with proper carrier generation, relaxation, and transfer.^51^ A *V*_OC_ of 1.34 V is obtained through our synergistic approach, which is attributed to the improved IRE and fluent carrier transport from PAL bulk. Through the dual-remedy of improving IRE and suppressing halide segregation in mixed halide perovskites, the PV parameters near ideality (Shockley–Queisser limit) can be approached.

Mitigating the notorious hysteresis phenomenon in Br-rich IPSCs is of utmost importance, and the hysteresis index can be measured quantitatively by the following relation (5)
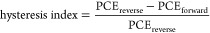
5

**Figure 5:**
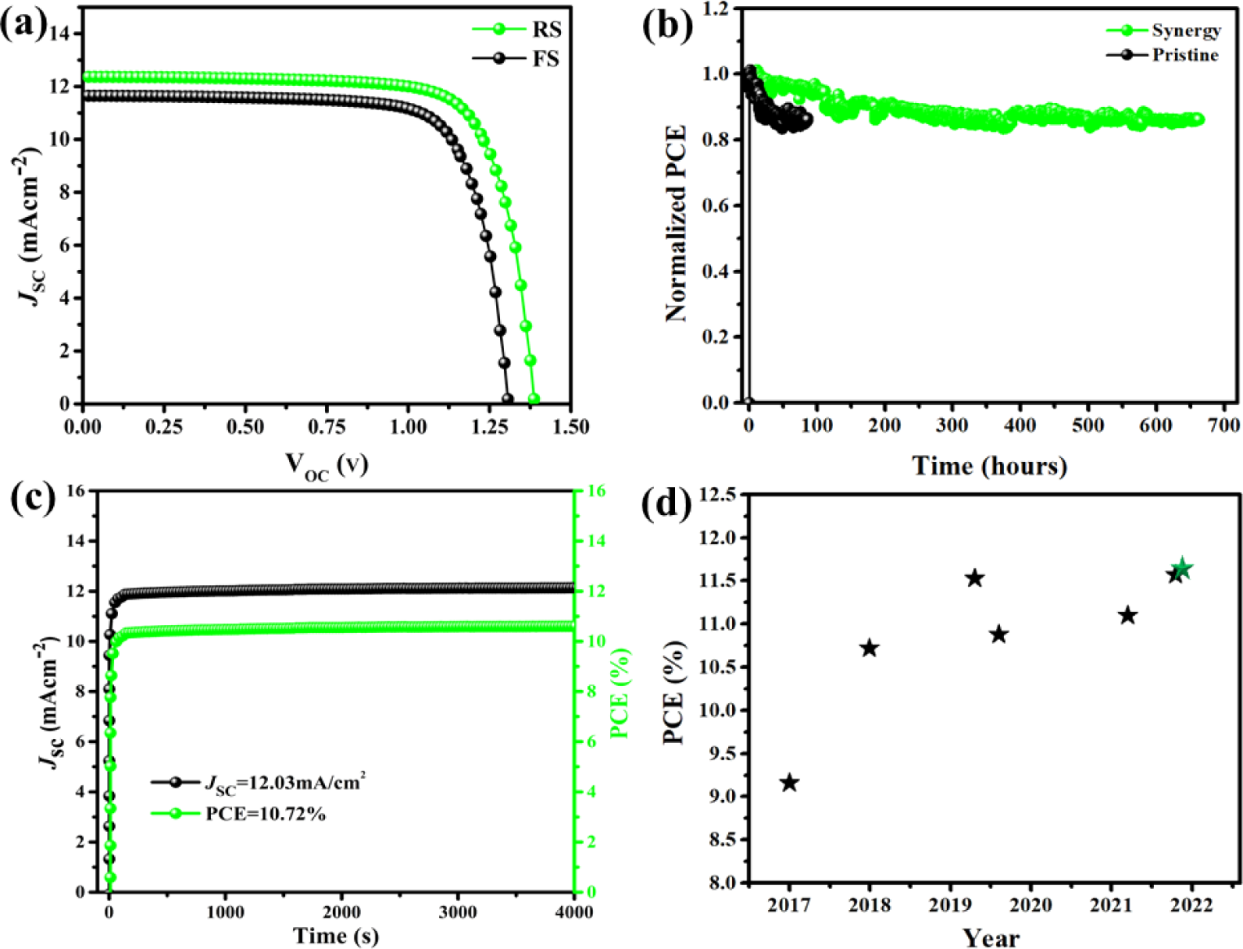
(a) *JV* hysteresis for synergy IPSC (b) stability against persistent illumination and (c) steady-state power output of synergy IPSC. (d) Statistics of PCE rise for CsPbIBr_2_-based PSCs.

As shown in Figure 5a, the hysteresis is mitigated from 15.5% (pristine) to 4.4% (synergy) IPSC, as shown in Figure S3b (Supporting Information). The ion migration within the wide band gap Br-rich PALs gives rise to phase segregation that leads to the formation of a greater injection barrier and intensifies hysteresis. Our synergistic approach enables the PAL to have suppressed phase segregation due to Cl inclusion (triple-anion)^49^ with fewer surface and deep traps, which in turn also derive smooth channels for carrier dynamics near CTLs and over the collection electrodes. We tested our optimized and pristine devices under constant 1 sun illumination (100 W cm^–2^), as shown in Figure 5b. The synergistically derived IPSC presents a stable device operation for up to 688 h (which is the highest for organic-free CsPbIBr_2_ IPSCs), as compared to the pristine IPSC, which is stable for only 116 h. We perform the steady-state performance of our optimized IPSC device at a bias of 1.13 and 0.85 V for synergy and pristine IPSCs, respectively. Figure 5c presents the figure of merit for SPO, as the optimized IPSC provides a constant photocurrent density of 12.03 mA cm^–2^ with a stabilized PCE of about 10.72% for 4000 s under ambient conditions, highlighting that our synergistic approach has the potential to synthesize high-performance PSCs. Figure 5d and Table S2 (Supporting Information) present the summary of previously reported CsPbIBr_2_ IPSCs, and our synergistically derived IPSC is the best performing device to date. We employ the durability test for our devices under room temperature ambience (25 °C, 35%-RH, and H_2_O < 0.1 ppm), and the stability plots for that are presented in Figure S7 (Supporting Information), which further testify that synergy IPSCs can comparatively endure harsh environments. The thermal stability of our device is also tested at different temperatures (27, 85, 100, and 120 °C) with constant heating for 120 min. Figures S8a–d statistically describe the stability of *JV* curves and PV parameters against various temperatures, and our IPSC device showed a very minute performance loss at 120 °C due to possible Ag egression into PAL through ETL.

**Figure 6:**
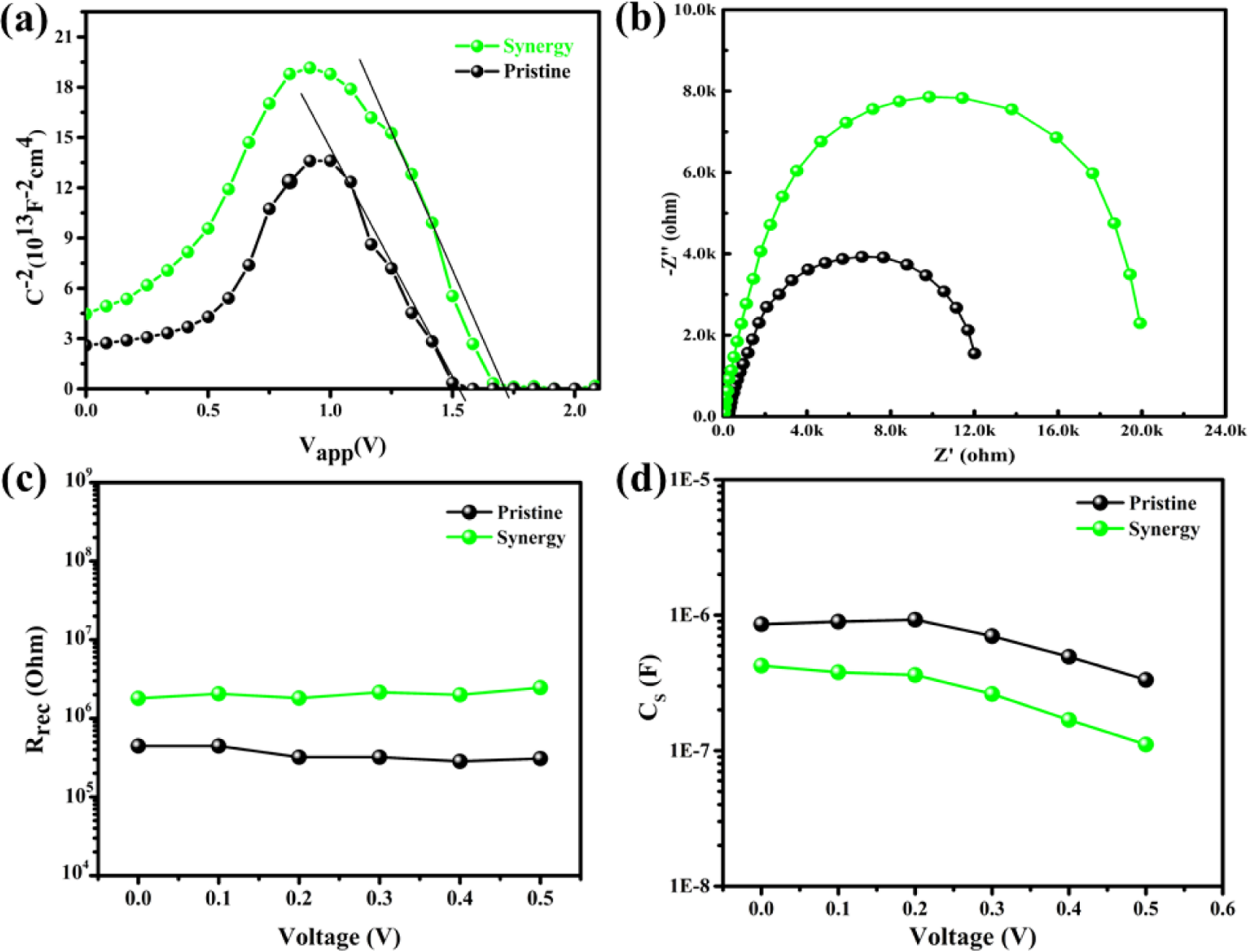
(a) Mott–Schottky analysis of pristine and optimized IPSCs, (b) EIS measurements for pristine and optimized IPSC devices, (c) *R*_rec_ as a function of bias, and (d) C_S_ as a function of bias.

We further investigate the origin of *V*_OC_ loss and improvement in FF through engaging in Mott–Schottky examination and calculating the built-in potential (*V*_bi_) to further verify PV device parameters. Figure 6a presents the estimation of (*V*_bi_); the optimized device attained comparatively enhanced *V*_bi_ at around 1.73 V (intercept with the *x*-axis), which is 230 mV higher than that of the pristine device (1.5 V). Holding the IPSC device in the dark, ensues with carrier diffusion, barrier capacitance, and *V*_bi_ after attaining the thermal equilibrium; due to the difference in the Fermi level across various functional layers within the whole IPSC stack.^52^ The applied reverse bias (*V*_app_) separates the Fermi levels from the state of equilibrium and hence screens out *V*_bi_ in the device heterojunction. The relation between bias voltage and capacitance can be given by the following Mott–Schottky relation (6)
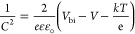
6where *C* represents the capacitance of the space charge region, *e* is the electronic charge, *V* is the applied potential, *k* is the Boltzmann constant, *T* is the absolute temperature, and *N*_D_ is the density of the donor.

Electrochemical impedance spectroscopy (EIS) is conducted to investigate the insight carrier dynamics of IPSC. We tested EIS for pristine and synergistic devices at a bias voltage of *V*_OC_, and the corresponding Nyquist-plot is presented in Figure 6b, with the equivalent model circuit in Figure S9a (Supporting Information). The shunt resistance (*R*_S_) is determined by the diameter of the semicircle and is generally related to carrier losses due to Shockley–Read–Hall or interfacial recombination. *R*_S_ for the synergistic IPSC is found to be larger than that for the pristine device, which represents the suppressed carrier recombination. The arc within the high-frequency region presents the process of carrier transportation from PAL through CTLs while combining transport resistance (*R*_tr_) and chemical capacitance (*C*_rec_), while the arc in the low-frequency region represents *R*_rec_ and *C*_rec_ at the CTL/PAL interface. Obviously, *R*_rec_ is higher (20.23 kΩ) for optimized IPSCs and lower (12.13 kΩ) for the pristine device, indicating suppressed recombination in optimized IPSCs, which is highly desired to get improved *V*_OC_. We further plotted the fitted *R*_rec_ against the bias voltage, as shown in Figure 6c; the synergistic IPSC presents a higher *R*_rec_ as compared to its pristine counterpart, depicting that the synergy technique has suppressed the possible carrier recombination, which enhanced the FF of the optimized device. Surface carrier accumulation was also suppressed through our synergistic approach, as Figure 6d shows the fitted results of capacitance (*C*_S_) as a function of bias voltage. The synergistic IPSC device presented lower *C*_S_ than the pristine device, which is also consistent with alleviated hysteresis in *JV* results.

Our synergistic approach derived a compact morphology with enhanced crystallization behavior, favorable to PAL’s physical properties. Consequently, we synthesize single-carrier devices (ITO/Nb_2_O_5_/CsI(PbBr_2_)_0.97_(ErCl_3_)_0.03_/PCBM/Ag) as shown in the inset of Figure S9b (Supporting Information) for the space-charge-limited current (SCLC) model to estimate trap-state density. The linear relationship of the *J*–*V* curve in the low-bias region of the voltage is an indication of Ohmic response, and the exceeding bias voltage of the kink point transforms it to a quadratic relationship, which is a sign of filled trap states. Therefore, the kink point between two regions is the trap-filled limit voltage (*V*_TFL_) that describes the trap-state density according to the underlying eq 7
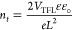
7where *L* is the PAL thickness (450 nm), ε is the dielectric constant, that is, about 8,^42^ ε_0_ is the vacuum permittivity, and “*e*” is the electronic charge. The *V*_TFL_ for pristine and optimized samples is 1.21 and 0.96 V as shown in Figure S6b (Supporting Information) with calculated trap densities of 1.68 × 10^16^ and 1.3 × 10^16^ cm^–3^, respectively. The optimized devices gained higher current density that explains the overall superior conductivity. Trap filling from the local increase in carrier density also gives rise to IRE, which suggests that optimized PALs have enormous potential to fill the carrier trapping sites at lower *V*_TFL_. The halide segregation phenomenon for triple-anion PALs is also dependent upon the trap-state densities and *V*_TFL_, as the carrier funneling bids the electrostatic driving force, causing halide segregation.^51^

## 3 Devised Agrivoltaics Scheme

The terminology of agrivoltaics (AgV) has arisen from the combination of agriculture and PVs. The formerly integrated greenhouses show that the benefits of the project are twofold, that is, the partial shading of plantation underneath AgV to reduce their water needs and the shelter to livestock from sunlight.^53^ The prospect of our proposed AgV project is to grow those vegetables and fruits which usually don’t grow under conventional sunlight irradiation and would be cultivated beneath an IPSC-enhanced AgV scheme. The harvested electrical energy can be utilized in production lines for cleaning and packaging of yields together with running of water pumps.

**Figure 7:**
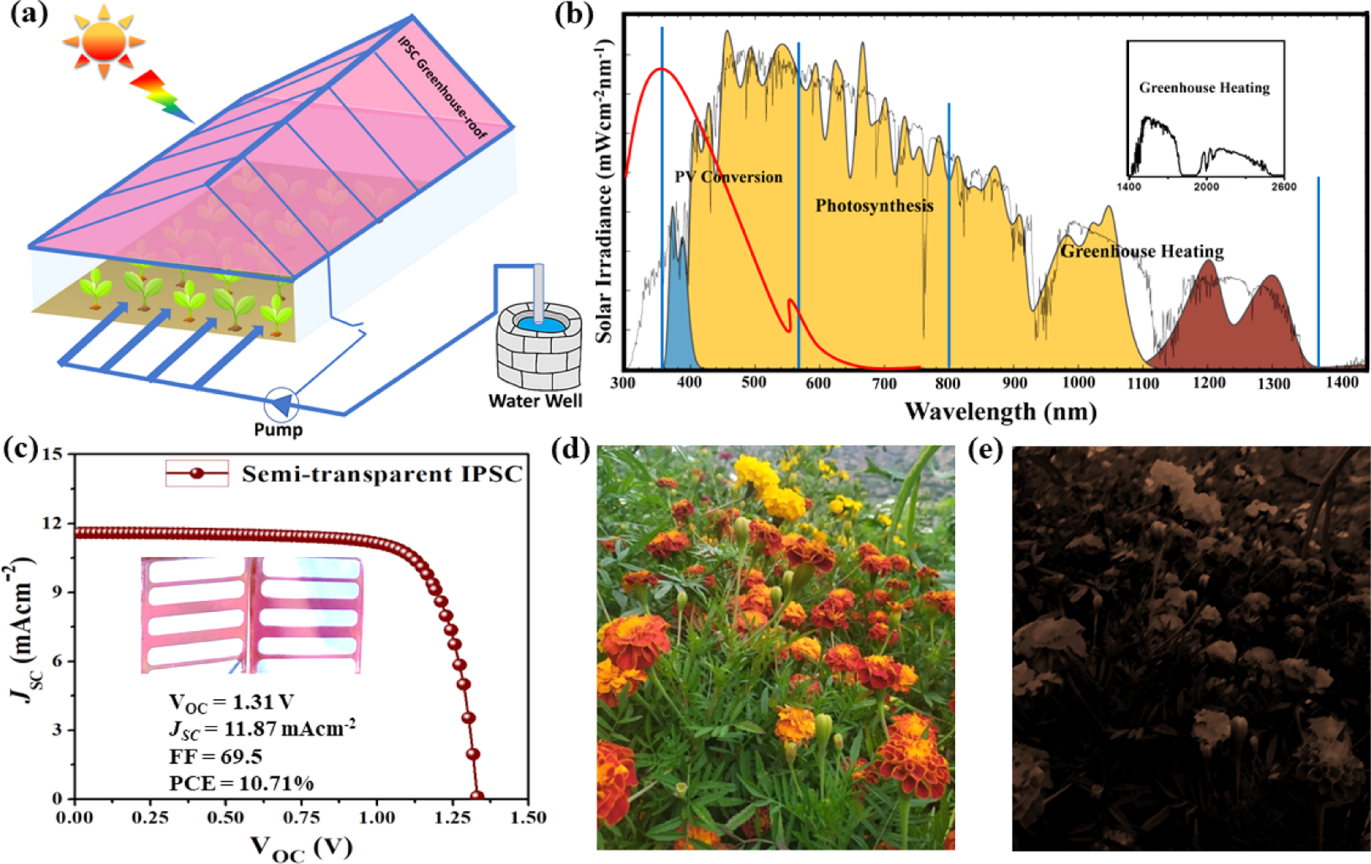
(a) Proposed AgV design, with a semi-transparent IPSC roof. (b) Distribution of photons to different regions of the visible portion of the solar spectrum. (c) *JV* curves for semi-transparent IPSC. Photograph of flower plantation (d) without IPSC shade (e) through semi-transparent IPSC.

The visible region of the solar spectrum is conventionally known as the photosynthetically active radiation, as for plant photosynthesis during greenhouse heating.^54^ Photosynthesis merely utilizes photons in the wavelength region of 560–800 nm and a part of the region of 400–550 nm wavelength.^55^ For our proposed AgV application (Figure 7a), the solar spectrum is divided into three portions/regions to balance PV conversion and photosynthesis with greenhouse heating through the target to utilize the maximum of the solar spectrum, as shown in Figure 7b. The region of short wavelengths, that is, 300–587 nm, comprises comparatively high-energy photons and is considered operative for PV conversion. The longer wavelength region of 587–800 nm, possesses a high photon-number density with low energy, which is suitable for plant photosynthesis and lighting. The third portion beyond the 800 nm wavelength is called the infrared region, ideal for greenhouse heating and temperature control.^56,57^ The photon number density in the three regions described above accounts for 19.2, 27.6, and 53.2%, respectively. We also synthesize a semi-transparent IPSC based on the same synergy but with high transparency of around 60% and the performance (PCE = 10.71%), as shown in Figure 7c with the inset picture of real semi-transparent IPSC. We optimize with a reduced thickness (0, 20, 40, and 60 nm) of the counter-electrode (Ag) so as to enhance the overall transparency of the semi-transparent IPSC device, as shown in Figure S9c. The devised mechanism, in which a semi-transparent IPSC-enhanced roof can serve as a photon filter, allows us to obtain considerable PV conversion and maintain normal plant photosynthesis with adequate photon management for greenhouse heating. The semi-transparent CsPbIBr_2_ IPSC is the best choice so as to have an absorption threshold of around 600 nm. Transmitted photons have longer wavelengths in near-infrared regions responsible for maintaining conventional plant photosynthesis and greenhouse heating at regular wavelengths.^58,59^Figure 7d,e, presents real-time pictures taken conventionally and through a semi-transparent IPSC device. Moreover, the inorganic photoactive PAL, with a wide band gap of 2.06 eV, has the potential to confine short radiation-enhanced photothermal conversion.^55,58^ In the phenomenon of photon–electron interaction, high-energy photons excite electrons from energy states lower than VBM and higher than CBM, while generating hot electron–hole pairs. The excitation energy is also converted to heat through non-radiative relaxation or phonon generation with the perovskite crystal lattice.^60,61^ Br-rich wide-band gap perovskites produce less heat energy than I-rich perovskites, which is good to harvest superb PV yields. Therefore, wide band gap semi-transparent IPSCs have the potential of higher PV conversion and less heat production in the short wavelength region of the solar spectrum, which will be a positive sign to manage temperature and to control the heat island effect within greenhouse-planted areas. Although the PCE for IPSCs is still farther away from its theoretical values, they can be potentially integrated as roofs into AgV projects, with the adjustment of transparency through counter-electrode replacement with TCO or some organic conducting polymers.^62,63^

## 4 Conclusions

In conclusion, a synergistic approach is presented to synthesize a highly efficient and stable organic-free IPSC with an improved PCE of 11.61%, which is also the highest for CsPbIBr_2_ based PSCs. The said synergy improved PAL’s morphology and crystallinity to micrometric grain sizes with enhanced IRE, obtained at a comparatively lower annealing temperature of 180 °C. Our optimized device received about a 60% increment in PCE compared to the pristine IPSC (7.28%) and retained 86% of its initial PCE after 688 h aging under persistent illumination. Moreover, semi-transparent IPSCs synthesized using the same synergistic approach can potentially be integrated into the AgV project. Our current approach has the integration potential for other compositional analogues of CsPbX_3_ to yield higher outputs.

## 5 Experimental Section

### 5.1 Chemical Availability

All the chemicals were used as received without further purification. Cesium iodide (CsI, 99.9985%) was purchased from Alfa Aesar and lead bromide (PbBr_2_, 99.999%) was purchased from Aladdin. DMSO (99.9%) was purchased from Sigma-Aldrich. Silver (Ag, 99.999%), nickel oxide (NiO, 99.9%), and niobium oxide (Nb_2_O_5_, 99.9%) were purchased from ZhongNuo Advanced Material (Beijing) Technology.

### 5.2 Solar Cell Fabrication

Substrates of ITO-coated glass (1.4 × 1.6 cm^2^) were cleaned with detergent, deionized water, acetone, and isopropanol for 15 min each in succession. Consequently, the NiO_*x*_ film (30 nm) as HTL was deposited onto the ITO/glass by electron beam evaporation (Angstrom Engineering, AMOD) through a metallic shadow-mask, with a deposition rate of ∼1 Å s^–1^ and a base pressure of <5 × 10^–6^ Torr. 30 nm thick NiO_*x*_–HTL film, then annealed at 300 °C in the air inside a tube furnace for 1 h. NiO_*x*_-coated substrates were transferred to a glovebox filled with N_2_ for CsPbIBr_2_ perovskite film deposition. The precursor solution for CsPbIBr_2_ was prepared by mixing PbBr_2_ (1 M, 367 mg) and CsI (1 M, 260 mg) in a solvent of DMSO (1 mL) with stirring for 3–4 h at room temperature and then filtering. Different molar ratios for the additive were prepared by dissolving ErCl_3_ in 1 mL of DMSO. An optimized amount of approximately. 0.03 mol % of ErCl_3_ was dissolved into 1 mL of DMSO through stirring at 60 °C for 4–5 h, and a minute amount of 50 μL was added into the original perovskite solution. For deposition of pristine and optimized films, pre-heated (95 °C) perovskite solution was spun-coated onto the NiO_*x*_-coated ITO substrates with a one-step dual-speed deposition program of 1500 rpm to 10 s and 4500 rpm to 60 s with post-annealing at 180 °C for 8–10 min. For semi-transparent IPSC, spin speeds were higher, that is, 2000 rpm for 10 s and 6000 rpm for 60 s, and the “Ag” counter electrode was optimized with thicknesses of 0, 20, 40, and 60 nm. Dark red colored uniform thin films were obtained, and after cooling down, the samples were then transferred out of the glovebox to the EB evaporation chamber for Nb_2_O_5_ (ETL) and Ag deposition. The Nb_2_O_5_ thin film, with a thickness of 60 nm, was deposited onto perovskite layers by EB evaporation at a deposition rate of approximately 1 Å s^–1^. A 100 nm thick Ag electrode was then deposited on top of the ETL through a metallic shadow-mask by EB evaporation at a base pressure of ∼5 × 10^–6^ Torr and a deposition rate of approximately 2 Å s^–1^. The active areas of the PSCs were 0.16 and 0.125 cm^–2^.

The DOSs of ErCl_3_-doped CsPbIBr_2_ (0, 10, 20, and 30%) were determined using first-principles DFT calculations. The projected augmented-wave method was employed to approximate exchange and correlation functionals, whereas the GGA of Perdew–Burke–Ernzerhof was utilized to deal with the electron–ion interactions with a cut-off energy of 400 eV. In addition, van der Waals correction of Grimme at the DFT-D3 level was incorporated throughout the calculations. The BZ sampling was performed by using a kpoints grid of 6 × 6 × 6 under the Monkhorst–Pack scheme. All the structures were relaxed until the force and energy criteria of 0.001 eV/Å 1 × 10^–6^ eV were met.

### 5.3 Characterizations

SEM images for morphology were characterized by field-emission SEM (SEM, Hitachi 8010 SU) at 5 kV as an acceleration voltage. Cu Kα radiation at 40 kV and 40 mA were used to operate Rigaku Miniflex 600 to test XRD spectra. A Bruker Dimension Icon instrument was utilized to test AFM images. UV–visible transmittance and absorption spectra were tested with the Shimadzu UV-2600 spectrometer. PL spectra, that is, both steady-state and time-resolved, were tested using a picosecond laser of wavelength 420 nm with a repetition rate between 10 and 40 MHz with the PicoQuant FluoTime 300 instrument. A solar simulator of AAA class (San-Ei-Electronic XES40S2) as a light source was used to test *J*–*V* curves of IPSCs. A certified reference cell (Konica Minolta AK-200) was used to calibrate the light intensity to 1-sun (100 mW cm^–2^). EQE spectra of IPSC devices were measured in the air with a lock-in amplifier coupled to a monochromator (Crowntech, QTest Station 2000).

## Supporting Information Available

Schematics of XPS; statistic grain-size distribution for pristine and synergistically derived CsPbIBr_2_ PALs; XRD spectra for pristine and optimized PALs; hysteresis calculation for the pristine IPSC; DFT calculation plots for total and partial DOSs; statistical distribution of 30 best devices for PV parameters and PCE; UPS plots for secondary electron cut-off; *J*–*V* curves taken after heating at a wide range of temperature for 120 min; PV parameters after heating the IPSC at different temperatures; equivalent electronic circuit for Nyquist-plot fitting; SCLC measurements to estimate *V*_TFL_ for pristine and synergistic IPSCs; transmittance for semitransparent IPSC with different layered thickness of the counter (Ag) electrode; equations for TRPL decay time estimation; formulae for derivation of EQE_EL_ and *J*_0_; PV parameters for the temperature, optimized additive concentration, and synergy approaches for IPSCs in current work; and comparison of PV parameters of CsPbIBr_2_-based solar cells (PDF)
