# Supplementary material for: Synergistic Approach toward Erbium-Passivated Triple-Anion Organic-Free Perovskite Solar Cells with Excellent Performance for Agrivoltaics Application
Source: ACS Appl Mater Interfaces. 2022 Jan 31;14(5):6894–905. doi: 10.1021/acsami.1c23476 (PMC8832393; doi:10.1021/acsami.1c23476)
Supplement: Supplementary file 1 — am1c23476_si_001.pdf [file am1c23476_si_001.pdf]

Supporting Information

A Synergistic Approach Towards Erbium–Passivated Triple Anion Organic–Free Perovskite Solar Cell with Excellent Performance for Agrivoltaics Application

M. Bilal Faheem[†], Bilawal Khan[‡], Chao Feng[†], Syed Bilal Ahmed[‡], Jiexuan Jiang[†], Mutee-Ur-Rehman[‡], W. S. Subhani[‡], M. U. Farooq[†], Jinlan Nie[*□], M. M. Makhlouf[*ɩ], and Quinn Qiao[*‖]

[†]Institute of Fundamental and Frontier Sciences, University of Electronic Science and Technology of China (UESTC), Chengdu 610054, P.R. China.

[‡]Department of Materials Science and Engineering, City University of Hongkong, Hongkong SAR, 999077, China.

[□]School of Physics, University of Electronic Science and Technology of China, Chengdu 610054, China.

[ɩ]Department of Sciences and Technology, Ranyah University College, Taif University, P.O. 11099, Taif 21944, Saudi Arabia.

[‖]Energy Generation and Storage Lab, Department of Mechanical and Aerospace Engineering, Syracuse University, Syracuse, NY 13244, United States.

Corresponding Authors.
[*]**Email.** quqiao@syr.edu (Q. Qiao)
[*]**Email.** m.makhlouf@tu.edu.sa (M. M. Makhlouf)
[*]**Email.** jinlannie@uestc.edu.cn (J. Nie)

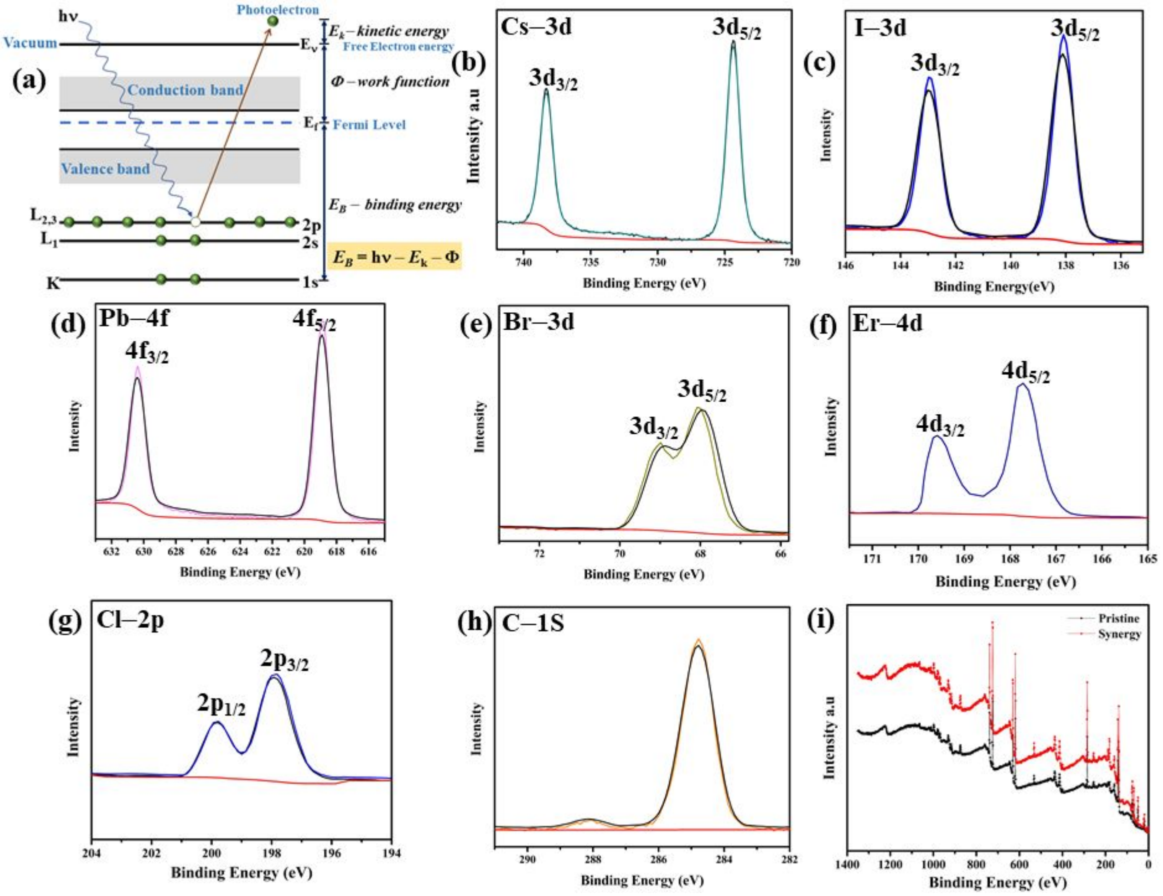

Figure S–1. Xray photoelectron spectroscopy (XPS) spectra against binding energy for (a) XPS-Schematics (b) Cs–3d (c) I–3d (d) Pb–4f (e) Br–3d (f) Er–4d (g) Cl–2p (h) C–1S (i) Survey spectra for Synergy and pristine PALs.

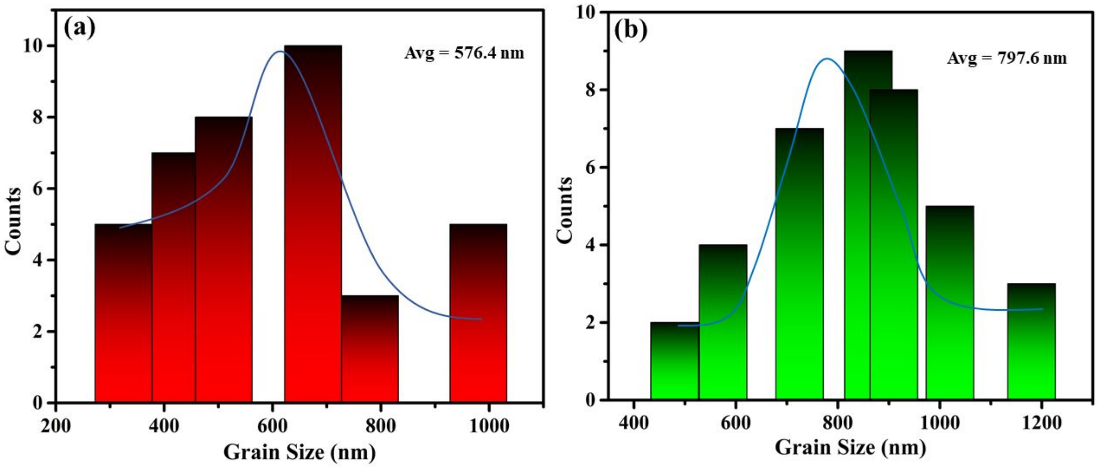

Figure S–2. Statistics of grain size distribution taken from SEM top-view images (a) Pristine (b) Synergy.

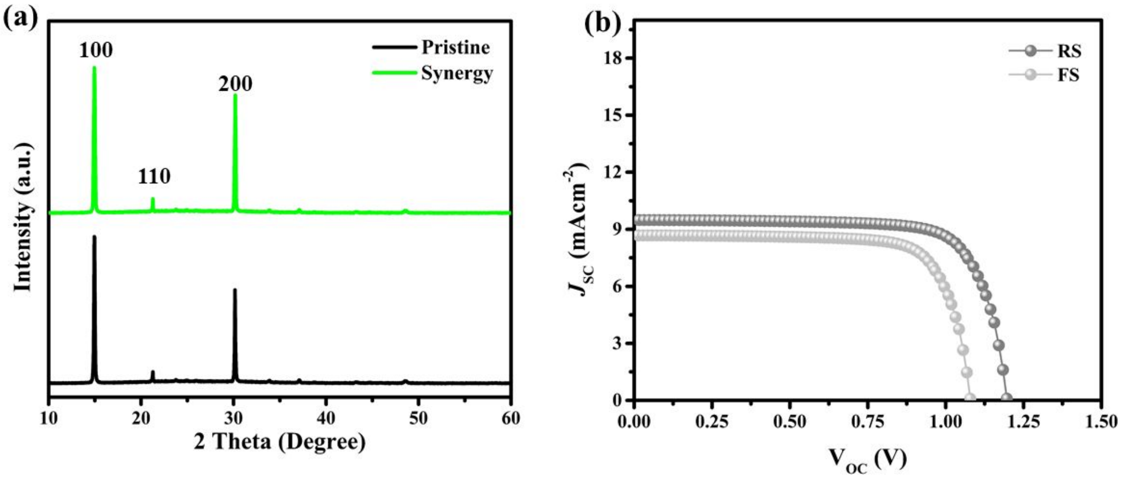

Figure S–3. (a) XRD spectra for Pristine and optimized PAL. (b) Hysteresis calculation for pristine IPSC.

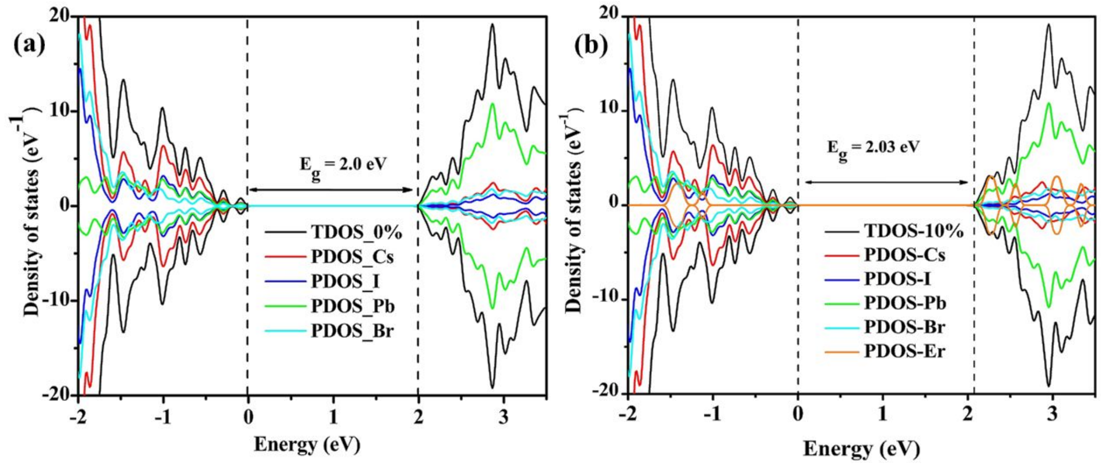

Figure S–4. Density functional theory (DFT) calculation plots presenting the density of states (DOS) of different perovskite constituent elements, for (a) TDOS and PDOS-0% (b) TDOS and PDOS-10% $ErCl_3$ doped $CsPbIBr_2$.

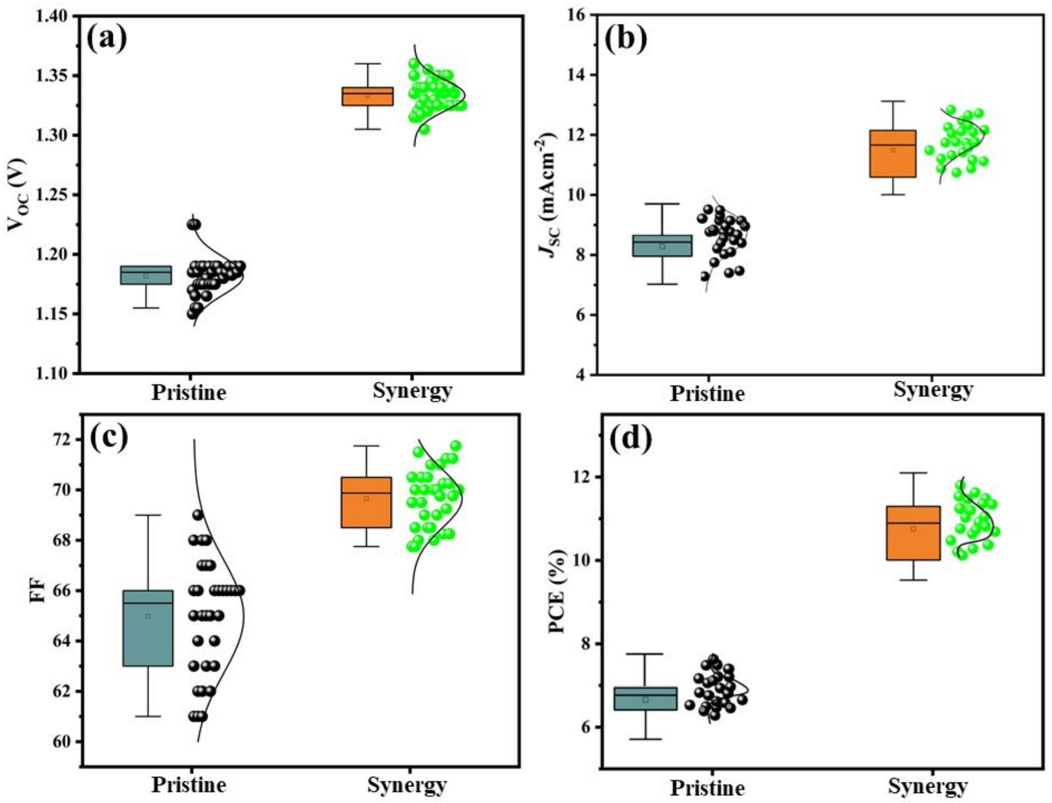

Figure S–5. Statistical distribution best performing devices for (a) V_{OC} (b) J_{SC} (c) FF (d) PCE.

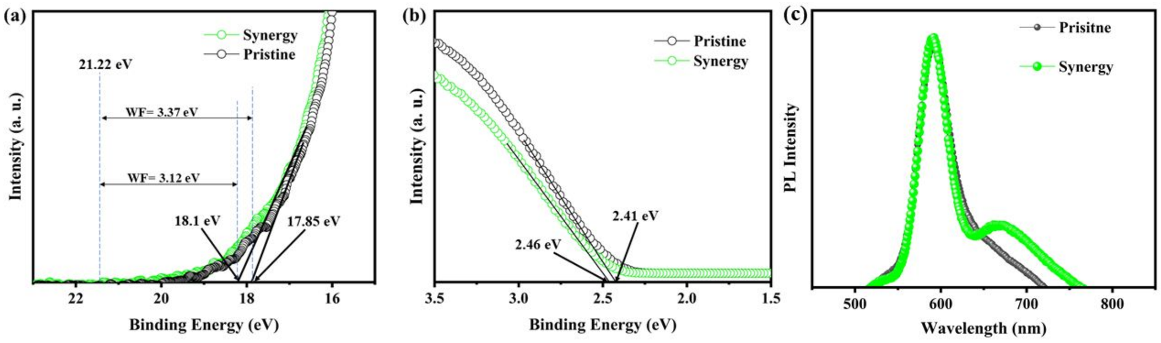

Figure S–6. UPS plots for (a) secondary electron cut-off, and (b) VBM energies against the intensity, (c) halide segregation for PAL yielding a V_{OC} of 1.34 V.

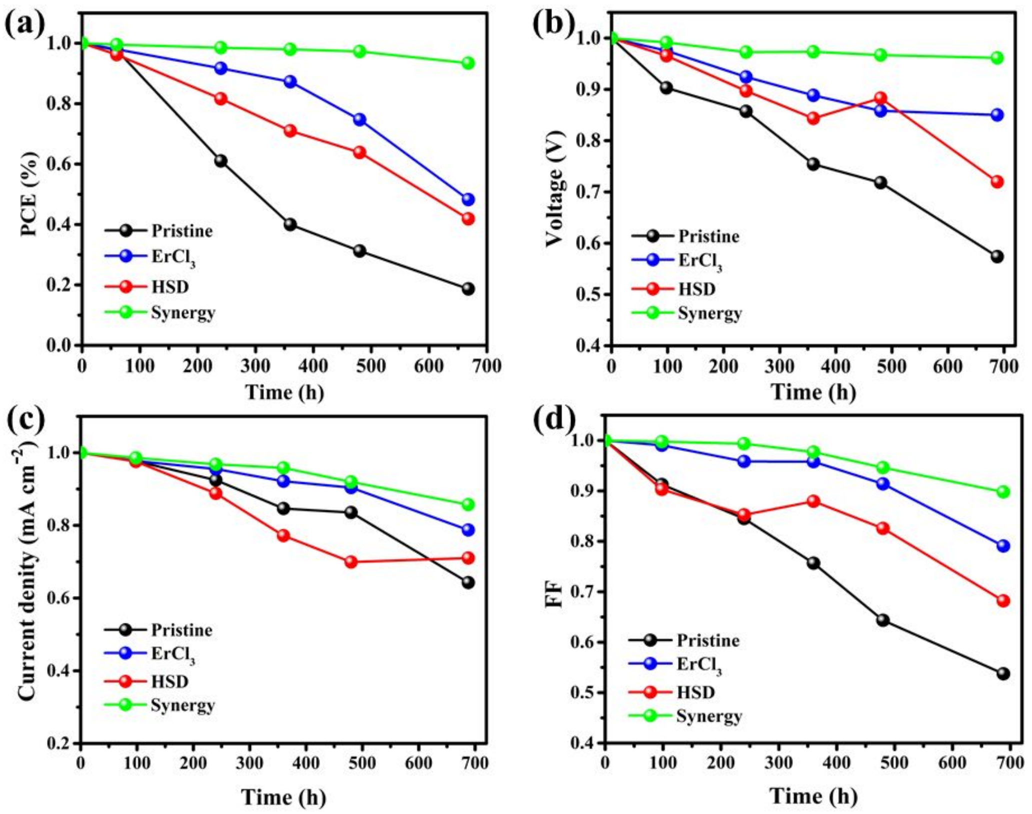

Figure S–7. Durability test of IPSC under room temperature ambience (25 °C, 35% RH, H_2O < 0.1 ppm) against time for (a) PCE (b) V_{OC} (c) J_{SC} (d) FF.

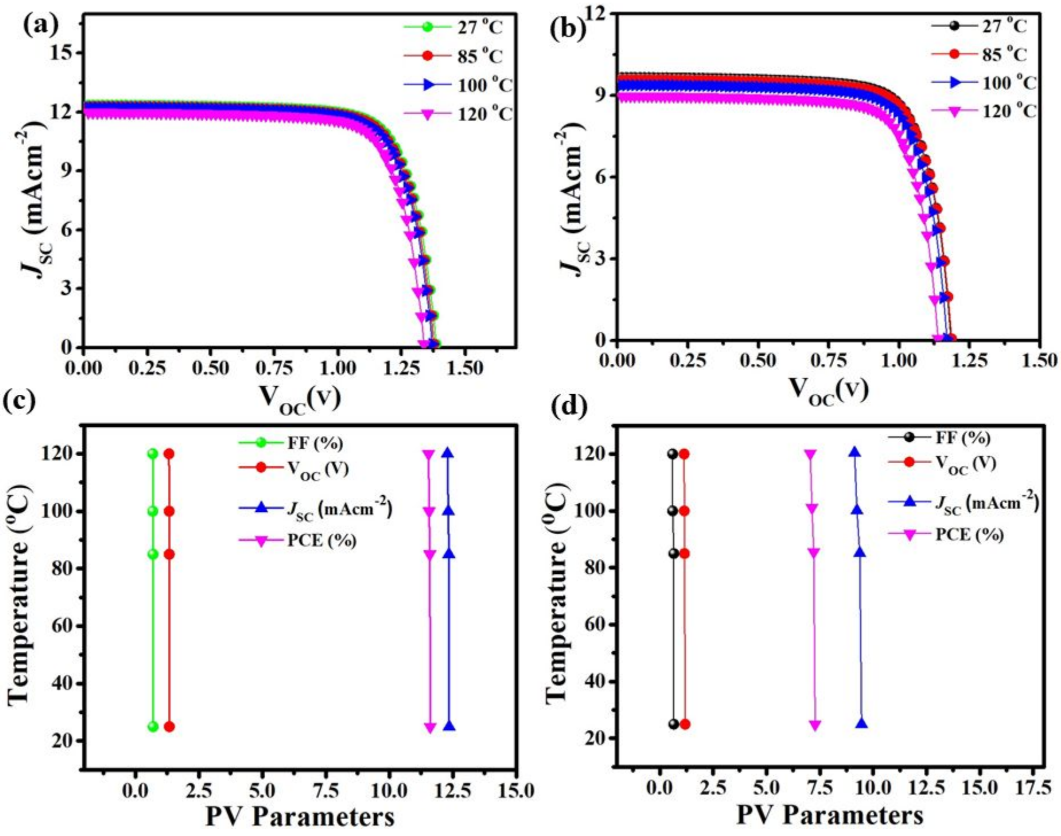

Figure S–8. *J*-V curves taken after heating at range of temperatures for 120 minutes (a) Pristine (b) Synergy. PV parameters after heating IPSC at different temperatures (c) Pristine (d) Synergy.

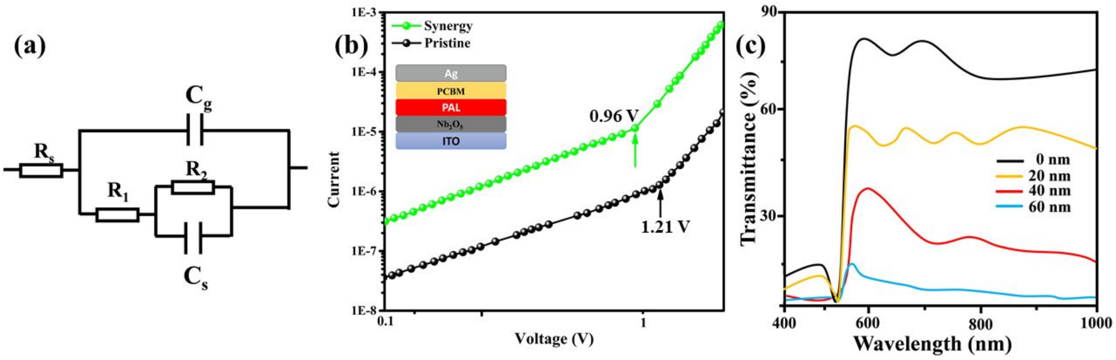

Figure S–9. (a) Equivalent electronic circuit for Nyquist–plot fitting (b) Space charge limited current (SCLC) measurements to estimate voltage trap-filled limits (V_{TFL}) for pristine and synergistic IPSCs (c) Transmittance of semitransparent IPSC with different layered thickness of counter (Ag) electrode.

UPS measurement.

$$VBM = 21.22 - (E_{cutoff} - E_{onset}) \quad \textbf{(S–1)}$$

$$Work\ Function\ (WF)\ = 21.22 - E_{cutoff} \quad \textbf{(S–2)}$$

EQE formulae.

$$EQE_{EL} = \frac{J_{0,rad}}{J_0} = \frac{J_{0,rad}}{J_{0,rad} + J_{0non-rad}} \quad \textbf{(S–3)}$$

$$J_0 = \frac{J_{0,rad}}{EQE_{EL}} = \frac{q}{EQE_{EL}} \int_0^{\infty} \varphi_{BB}(\lambda) \cdot \mathrm{EQE_{PV}}(\lambda) \cdot d\lambda \quad \textbf{(S–4)}$$

TRPL measurement.

The TRPL decay time and amplitudes are obtained using an exponential Equation (S–5);[1,2]

$$f(x) = \sum_i A_i \exp\,(-t/\tau_i) + K \quad \textbf{(S–5)}$$

where τ_i is the decay time, Ai is the decay amplitude and K is a constant for the baseline offset. To understand the recombination mechanism of the perovskite thin films on different substrates, the recombination kinetics was modelled over a range of excitation intensities using the following Equation (S–2);[1,2]

$$-\frac{dn}{dt} = An + Bn^2 + Cn^3 \quad \textbf{(S–6)}$$

where t is the time and n is the photogenerated excess carrier density. The physical interpretations of these three terms are (i) the first-order decay rate is due to the trap-mediated (Shockley-Hall-Read) recombination at low injection condition; (ii) the second-order decay rate is due to the non-geminate/free carrier recombination at high injection; and (iii) the third order decay rate is for the Auger recombination. When glass/pristine-$CsPbIBr_2$, glass/hot-solution- $CsPbIBr_2$ and glass/synergy- $CsPbIBr_2$ are analyzed, the tremendous

second-order decay rate is observed for the photogenerated carrier easy injection from perovskite to the NiO HTL. Based on the above analysis, the PL decay time obtained by bi-exponential function is used to fit the PL decay time.

Table S–1. PV parameters for temperature, optimized additive concentration, and synergy approaches for IPSCs.

Temperature (°C)	V_{OC} (V)	J_{SC} (mAcm^{-2})	FF (%)	PCE (%)
**Pristine**	1.19	9.46	66	7.28
75	1.21	10.61	66	8.37
85	1.23	10.93	67	8.91
**95**	**1.26**	**11.48**	**68**	**9.48**
**Concentration (mol %)**				
0.01	1.22	11.17	67	9.10
0.02	1.27	11.54	68.5	10.14
**0.03**	**1.31**	**11.82**	**69**	**10.61**
0.04	1.16	9.8	62	6.92
0.05	1.10	8.72	59	5.52
**Synergy**	**1.34**	**12.36**	**70.5**	**11.61**

Table S–2. Previous reports of $CsPbIBr_2$ PSCs.

PSC Configuration	Annealing Temperature (C)	V_{OC} (V)	PCE (%)	SPO	Reference
FTO/TiO_2/$CsPbIBr_2$/Spiro/Au	320	1.227	8.02	–	[3]
FTO/c-TiO_2/$CsPbIBr_2$/Carbon	280	1.245	9.16	–	[4]
FTO/SnO_2/$CsPbIBr_2$/Spiro/Au	250	1.24	11.1	10.1	[5]
FTO/TiO_2/$SmBr_3$/$CsPbIBr_2$/Spiro /Au	225	1.17	10.88	9.96	[6]
FTO/TiO_2/CsBr/$CsPbIBr_2$/Carbon	280	1.261	10.71	–	[7]
FTO/TiO_2/$CsPbIBr_2$/Spiro/Ag	250	1.25	10.43	–	[8]
ITO/SnO_2/$CsPbIBr_2$/Spiro/Ag	250	1.267	9.86	8.78	[9]

FTO/TiO_2/$CsPbIBr_2$/Spiro-OMeTAD/Ag	225	1.21	10.4	7.65	[10]
FTO/c-TiO_2/$CsPbIBr_2$/NP-GO/carbon	260	1.29	10.95	10.1	[11]
FTO/SnO_2/$CsPbIBr_2$-PEI/NiO/Ag	280	1.25	11.30	–	[12]
ITO/TiO_2/$CsPbIBr_2$/P3HT:PC_{61}BM/Carbon	160	1.31	11.54	10.63	[13]
**ITO/NiO/CsI$(PbBr_2)_{0.97}(ErCl_3)_{0.03}$/$Nb_2O_5$/Ag**	**180**	**1.34**	**11.61**	**10.72**	**This work**

References.

1. Manser, J. S; Kamat, P.V., Band Filling With Free Charge Carriers in Organometal Halide Perovskites. *Nat. Photon.* **2014**, *8*, 737-743.

2. Tosun. B.S; Hillhouse. H. W., Enhanced Carrier Lifetimes of Pure Iodide Hybrid Perovskite via Vapor-Equilibrated Re-Growth (VERG) J. Phys. Chem. Lett. **2015,** 6, 2503-2508.

3. Li, W.; Rothmann, M. U.; Liu, A.; Wang, Z.; Zhang, Y.; Pascoe, A. R.; Lu, J.; Jiang, L.; Chen, Y.; Huang, F., Phase Segregation Enhanced Ion Movement in Efficient Inorganic $CsPbIBr_2$ Solar Cells. *Adv. Energy Mater.* **2017,** *7* (20), 1700946.

4. Zhu, W.; Zhang, Q.; Chen, D.; Zhang, Z.; Lin, Z.; Chang, J.; Zhang, J.; Zhang, C.; Hao, Y., Intermolecular Exchange Boosts Efficiency of Air-Stable, Carbon-Based All-Inorganic Planar $CsPbIBr_2$ Perovskite Solar Cells to Over 9%. *Adv. Energy Mater.* **2018,** *8* (30), 1802080.

5. Zhang, W.; Xiong, J.; Li, J.; Daoud, W. A., Seed-Assisted Growth for Low-Temperature-Processed All-Inorganic $CsPbIBr_2$ Solar Cells with Efficiency over 10%. *Small* **2020,** *16* (24), 2001535.

6. Subhani, W. S.; Wang, K.; Du, M.; Wang, X.; Liu, S., Interface-Modification-Induced Gradient Energy Band for Highly Efficient $CsPbIBr_2$ Perovskite Solar Cells. *Adv. Energy Mater.* **2019,** *9* (21), 1803785.

7. Zhu, W.; Zhang, Z.; Chai, W.; Zhang, Q.; Chen, D.; Lin, Z.; Chang, J.; Zhang, J.; Zhang, C.; Hao, Y., Band Alignment Engineering Towards High Efficiency Carbon-Based Inorganic Planar $CsPbIBr_2$ Perovskite Solar Cells. *ChemSusChem* **2019,** *12* (10), 2318-2325.

8. Sun, H.; Yu, L.; Yuan, H.; Zhang, J.; Gan, X.; Hu, Z.; Zhu, Y., $CoCl_2$ as Film Morphology Controller for Efficient Planar $CsPbIBr_2$ Perovskite Solar Cells. *Electrochimica Acta* **2020,** *349*, 136162.

9. Guo, Y.; Yin, X.; Liu, J.; Que, W., Highly Efficient $CsPbIBr_2$ Perovskite Solar Cells with Efficiency Over 9.8% Fabricated Using a Preheating-Assisted Spin-Coating Method. *J. Mater. Chem. A* **2019,** *7* (32), 19008-19016.

10. Liu, P.; Yang, X.; Chen, Y'; Xiang, H.; Wang, W.; Ran, R.; Zhou, W.; Shao, Z.,

Promoting the Efficiency and Stability of $CsPbIBr_2$-Based All- Inorganic Perovskite Solar Cells through a Functional Cu^{2+} Doping Strategy. ACS Appl. Mater. Interfaces **2020,** 12, 23984−23994.

11. Du, J.; Duan, J.; Yang, X.; Duan, Y.; Zhou, Q.;Tang, Q.;, p-Type Charge Transfer Doping of Graphene Oxide with $(NiCo)_{1-y}Fe_yO_x$ for Air-Stable, All-Inorganic $CsPbIBr_2$ Perovskite Solar Cells, Angew. Chem. Int. Ed. **2021,** 60, 10608 –10613.

12. Gao, B; Meng, J.; Highly Stable All-Inorganic $CsPbIBr_2$ Perovskite Solar Cells with 11.30% Efficiency Using Crystal Interface Passivation. ACS Appl. Energy Mater. **2020,** *3*, 8249−8256.

13. Wang, D.; Li, W.; Li, R.; Sun, W.; Wu, J.; Lan, Z*;. High-Efficiency Carbon-Based $CsPbIBr_2$ Solar Cells with Interfacial Energy Loss Suppressed by a Thin Bulk-Heterojunction Layer. Sol. RRL. **2021,** 5, 2100375.
